# Frequent downregulation and loss of WWOX gene expression in human hepatocellular carcinoma

**DOI:** 10.1038/sj.bjc.6602023

**Published:** 2004-07-13

**Authors:** S-W Park, J Ludes-Meyers, D B Zimonjic, M E Durkin, N C Popescu, C M Aldaz

**Affiliations:** 1 Laboratory of Experimental Carcinogenesis, Center for Cancer Research, National Cancer Institute, Bethesda, MD 20892-4262, USA; 2 Department of Carcinogenesis, The University of Texas MD Anderson Cancer Center, Smithville, TX 78957, USA

**Keywords:** WWOX gene expression, tumour suppressor gene, hepatocellular carcinoma, chromosome rearrangements, fragile sites

## Abstract

The WWOX (WW-domain containing oxidoreductase) is a candidate tumour suppressor gene spanning the same chromosome region, 16q23, as the second most common fragile site (FS), FRA16D. Deletions detected by comparative genomic hybridisation (CGH) and loss of heterozygosity at microsatellite markers on chromosome 16q are common in many human cancers including hepatocellular carcinoma (HCC). The development of human HCC is closely associated with exposure to oncogenic viruses and chemical carcinogens, agents known to frequently target common FS. We examined the status of WWOX genomic DNA, RNA and protein in 18 cell lines derived from human HCC and found recurrent alterations of the gene. Loss of DNA copy-number confined to band 16q23 was detected by CGH in several cell lines. Although homozygous deletions of the WWOX gene were not detected, WWOX mRNA expression was absent or lower in 60% of cell lines. The occurrence of aberrant WWOX reverse transcription–PCR products with deletion of exons 6–8 correlated significantly with altered WWOX expression. All of the cell lines showing mRNA downregulation had a decreased or undetectable level of WWOX protein as demonstrated by Western blotting with antibody to WWOX. Furthermore, 13 out of the 18 cell lines expressed decreased levels or no WWOX protein when compared with normal liver. These results show that WWOX gene is frequently altered in HCC and raise the possibility that this gene is implicated in hepatocarcinogenesis.

Hepatocellular carcinoma (HCC) is one of the most common cancers worldwide and has a high mortality rate, as many tumours are asymptomatic until the later stages (Thorgeirsson and Grisham, 2002). Infection with hepatitis B and C viruses (HBV and HCV) and exposure to the dietary carcinogen, aflatoxin account for almost 80% of HCC. The progression to HCC is a stepwise process evolving over a long period of time through distinct stages associated with cumulative genetic lesions. Despite the abundance of recurrent genomic alterations identified over the past several years, relatively few genes have been found to be affected by deletion, amplification or mutation in HCC (Thorgeirsson and Grisham, 2002). The identification of genes that contribute to the development of HCC thus continues to be critical to our understanding of molecular mechanisms of hepatocarcinogenesis and the treatment of liver cancer. Chromosomal translocations, inversions, deletions, and amplifications contribute to the pathogenesis of human cancer by affecting the expression of genes involved in regulating cell growth (Bishop, 1987; Mitelman *et al*, 1997; Hanahan and Weinberg, 2000). Characterisation of recurrent chromosome changes in human HCC at the molecular level resulted in the identification of genes implicated in neoplastic development. From one region of recurrent deletion on the short arm of chromosome 8 in HCC, a novel gene, DLC-1, was cloned and recently shown to operate as a tumour suppressor gene in different types of solid tumours including HCC (Yuan *et al*, 2003a; Zhou *et al*, 2004). Alterations have been detected in several known genes, including deletion and downregulation of the tumour suppressor gene FHIT at 3p, amplification of the EMS1 oncogene at 11q, and high level amplification of the SMAD5 gene at 5q; these may be pathologically relevant to HCC and might be useful markers for early detection and prognosis of the disease (Yuan *et al*, 2000, 2003b; Zimonjic *et al*, 2003). The FHIT, EMS1 and SMAD5 genes are located at aphidicolin (apc)-inducible common fragile sites (FSs) FRA3B, FRA11B and FRA5C, respectively. The role of FSs in cancer did not receive much attention until relatively recently, when it was discovered that genomic alterations in cancer cells are frequently the consequence of breakage at FSs. Breakage at FSs inflicted by chemical and physical carcinogens and oncogenic viruses could result in chromosome translocations, amplification of proto-oncogenes or deletion of tumour suppressor genes (Smith *et al*, 1998; Huebner and Croce, 2001; Popescu, 2003).

WWOX (WW-domain containing oxidoreductase), a candidate tumour suppressor gene, spans over 1 Mb of DNA at 16q23.3–24.1, the location of FRA16D, the second most active FS in human genome (Glover *et al*, 1984; Bednarek *et al*, 2000, 2001; Ludes-Meyers *et al*, 2003). As shown by many comparative genomic hybridisation (CGH) and microsatellite analyses of primary and metastatic human HCC and HCC-derived cell lines, loss of DNA copy-number and LOH on the long arm of chromosome 16 are recurrent alterations (Thorgeirsson and Grisham, 2002). Using a panel of microsatellite markers the minimal region of LOH in HCC was defined at 16q23.1–24.1 in one study and localised to intron 8 of the WWOX gene in another (Balsara *et al*, 2001; Yakicier *et al*, 2001). Chromosomal regions that are frequently deleted in cancer are thought to be the loci of tumour suppressor genes, whose loss or inactivation allows unrestricted cell proliferation. Given the high frequency of LOH and loss of DNA copy-number on 16q23–24 in human HCC regardless of their geographic derivation or association with different etiological factors, we sought to find out whether alterations of the WWOX gene at FRA16D are recurrent in HCC and to determine their nature and relevance to hepatocarcinogenesis.

## MATERIALS AND METHODS

### HCC cell lines

The HCC cell lines used in this study have been previously characterised for their cytogenetic constitution and viral profile (Yuan *et al*, 2000, 2003b; Zimonjic *et al*, 2003). The cells were cultured in DMEM/F12 medium supplemented with 10% foetal bovine serum and antibiotics. Five cell lines, SK-Hep-1, 7703, HepG2, Huh-7, HLF and HLE are negative for HBV.

### Northern blot analysis

Total RNA was extracted from cell lines and analysed by Northern blotting as described earlier (Zimonjic *et al*, 2003). The blot was hybridised to a 1490-bp WWOX cDNA probe (nt 62–1551 of GenBank AF211943), synthesised by RT–PCR using the forward primer (5′-GAGTTCCTGAGCGAGTGGAC-3′ located on exon 1) and the reverse primer (5′-CCCCAGGAATTCCCTGCTT-3′ located on exon 9). The gel-purified probe was labelled with [*α*-^32^P]dCTP, and hybridisation and washing of the blots were performed as previously described (Zimonjic *et al*, 2003). The blot was exposed to autoradiographic film (Kodak, Rochester, NY, USA) for 2 days, the autoradiogram was scanned on an HP Scanjet 4400c (Hewlett Packard, Palo Alto, CA, USA). The WWOX probe was stripped from the blot and the membrane was re-hybridised with the human *β*-actin cDNA control probe.

### Reverse transcription–PCR analysis

First-strand cDNA was prepared from 5 *μ*g of total RNA using the Superscript first strand synthesis system (Invitrogen, Carsbad, CA, USA). After reverse transcription, the cDNA was used as a template for PCR amplification of the human WWOX cDNA. The PCR mixture contained 2 *μ*l of the first-strand reaction, 100 *μ*M dNTPs, 1.5 mM MgCl_2_, 1/10 volume of 10 × PCR buffer and 10 pmol each of the forward and reverse primers indicated above and 1.25 U AmpliTaq Gold DNA polymerase (Perkin-Elmer, Foster city, CA, USA). Amplification was carried out as follows: an initial denaturation for 5 min at 95°C followed by 35 cycles of 94°C for 1 min, 57°C for 1 min, 72°C for 1 min 30 s, and a terminal extension for 10 min at 72°C, yielding a 1490-bp product. As a control, human *β*-actin was amplified using the forward primer (5′-GTGGGGCGCCCCAGGCACCA-3′) and the reverse primer (5′-CTCCTTAATGTCACGCACGATTTC-3′). The PCR products were analysed by gel electrophoresis on 2% agarose gels, stained with 20 *μ*g ml^−1^ of ethidium bromide, and visualised by UV transillumination.

### Southern blot analysis

Genomic DNA was extracted from the HCC cell lines by a modification of the original method (Laird *et al*, 1991), and normal human genomic DNA was purchased from Promega (Madison, WI, USA). In total, 5 *μ*g aliquots of each genomic DNA were digested with the restriction endonuclease *EcoR*I (New England Biolabs, Beverly, MA, USA) for 4 h and were electrophoresed on a 1% agarose gel in 1 × TAE. Gels were transferred onto Hybond N+ membranes (Amersham Pharmacia, Piscataway, NJ, USA) and were UV crosslinked and hybridised with the ^32^P-labelled WWOX cDNA probe, as described above. After washing, the membranes were exposed overnight to autoradiographic film (Kodak, Rochester, NY, USA) at −80°C. Relative intensities of the bands were normalised using the *β*-actin cDNA probe as an internal reference control.

### Western blot analyses

Total cell protein lysates were made using RIPA buffer (50 mM Tris pH 7.5, 150 mM NaCl, 0.5% sodium deoxycholate, 1% Triton X-100, 0.1% SDS) containing protease inhibitor cocktail (Roche, Mannheim, Germany). HCCs were washed twice with PBS and then lysed in RIPA buffer at 4°C for 1 h, centrifuged at 10 000 **g** for 10 min to remove cell debris. Human normal liver protein extracts were prepared from pulverised frozen tissue. In addition, normal liver protein extract was purchased from ProSci Inc (Poway, CA, USA). For Western blotting, 50 *μ*g of total protein was separated by 12.5% SDS–PAGE and transferred onto PVDF membranes (Millipore, Billerica, MA, USA). Each gel had a lane containing a protein lysate from a cell line stably transfected with a WWOX expression vector (Peo/WWOX) as a reference for standardisation of independent experiments (Ludes-Meyers *et al*, 2003). Immunodetection was performed using Protein Detector™ (KPL, Gaithersburg, MD, USA) Western blotting reagents as described by the manufacturer. WWOX protein was detected using affinity purified anti-WWOX polyclonal primary antibodies (Ludes-Meyers *et al*, 2003) and HRP conjugated anti-rabbit secondary antibody (KPL, 1 : 5000) followed by chemiluminescence autoradiography. Actin was detected using monoclonal anti-actin antibody (ICN biomedicals, Burlingame, CA, USA, 1 : 1000) and HRP conjugated anti-mouse secondary antibody (KPL, 1 : 5000). Quantitation of Western blots was performed using a Kodak digital science Image Station 440CF. For each autoradiograph the WWOX values were first normalised to actin (WWOX/actin) and then standardised to the WWOX reference. The standardised WWOX/actin for each HCC was then divided by the standardised WWOX/actin value of normal liver to obtain HCC WWOX/Normal WWOX.

### WWOX immunohistochemistry

Tissue sections from five normal liver samples and five HCC were stained with WWOX antibody. Prior to anti-*WWOX* immunostaining, endogenous peroxidase activity was blocked with 3% H_2_O_2_ in water for 10 min. Heat-induced epitope retrieval was performed with 1.0 mM EDTA buffer pH 8.0 for 10 min in a microwave oven followed by a 20-min cooldown. In order to block nonspecific antibody binding, tissue sections were incubated with 10% goat serum in PBS for 30 min. Primary polyclonal rabbit WWOX antibody (140 *μ*g ml^−1^) was used at a 1 : 50 dilution overnight at 4°C. This was followed by incubation with Envision HRP labelled polymer conjugated to goat-anti-rabbit immoglobulins (Dako) for 30 min at room temperature. Staining development was performed with DAB with timed monitoring using a positive control sample. The slides were then counterstained with haematoxylin, dehydrated, cleared and mounted.

### Mutation screening of the WWOX gene

Genomic DNA isolated from the HCC cell lines was used to amplify all exons of the WWOX gene. PCR amplifications were performed using the primers described earlier (Bednarek *et al*, 2000). The reactions contained 50 ng of genomic DNA, 100 *μ*M dNTPs, 1.5 mM MgCl_2_, 1/10 volume of 10 × PCR buffer, 10 pmol of each primer, and 1.0 U Taq DNA polymerase (Promega, Madison, WI, USA), in a final volume of 20 *μ*l. The PCR conditions and the SSCP analysis of the products were performed as described earlier (Park *et al*, 2003).

## RESULTS

### Comparative genomic hybridisation profile of chromosome 16

**Figure 1:**
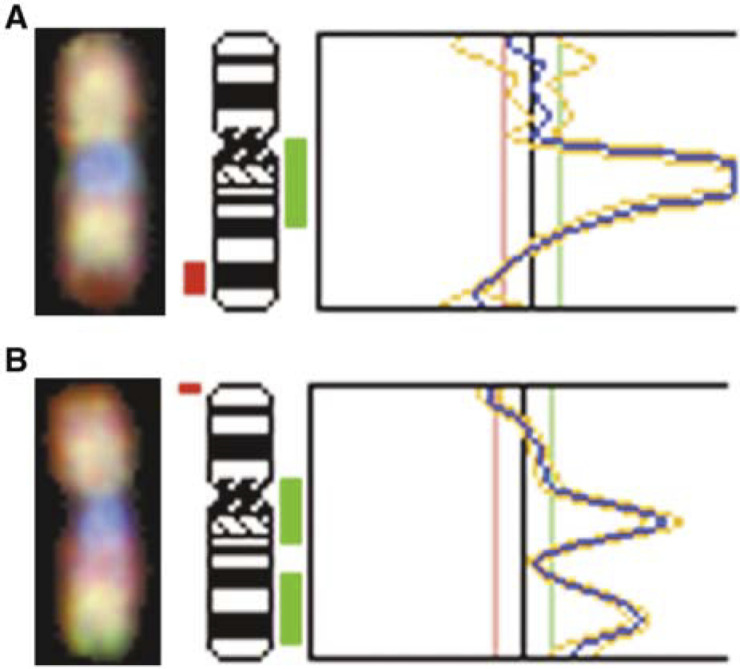
Comparative genomic hybridisation profile for chromosome 16 in HLE (**A**) and HepG2 (**B**) HCC cell lines. HLE cells exhibit loss of DNA copy-number at region 16q23 (red) and HepG2 cells have gain of DNA copy-number at two sites on the long arm of chromosome 16 (green).

Consistent with CGH analyses of many primary tumours and metastatic HCC, our CGH profile of HCC cell lines showed recurrent loss of DNA copy-number on chromosome 16q (Zimonjic *et al*, 1999). Furthermore, in the PLC/PRF/5, SK-Hep-1, HLF, HLE, and SNU387 cell lines, loss of DNA copy-number was confined to a single band, 16q24, corresponding to the location of the WWOX gene. In the Hep40 cell line the whole chromosome 16 was over-represented and in HepG2 gain on the long arm was detected. A representative profile of chromosome 16 in HLE and HepG2 cells, showing loss at 16q24 and gain of 16q, respectively, is presented in Figure 1

.

### Transcriptional expression of WWOX gene

**Figure 2:**
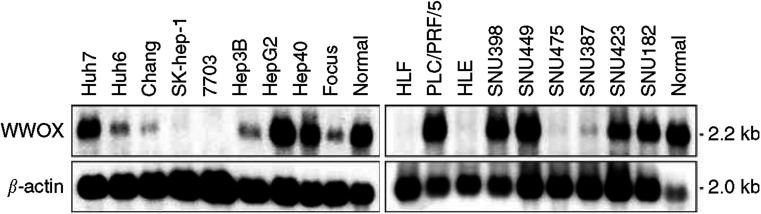
Northern blot analysis of total RNA from HCC cell lines. The WWOX cDNA probe detected a 2.2-kb transcript. No signal was detected in the SK-Hep-1, 7703, HLF and HLE cell lines. The blots were re-hybridised to a *β*-actin probe as a control for RNA loading.

**Figure 3:**
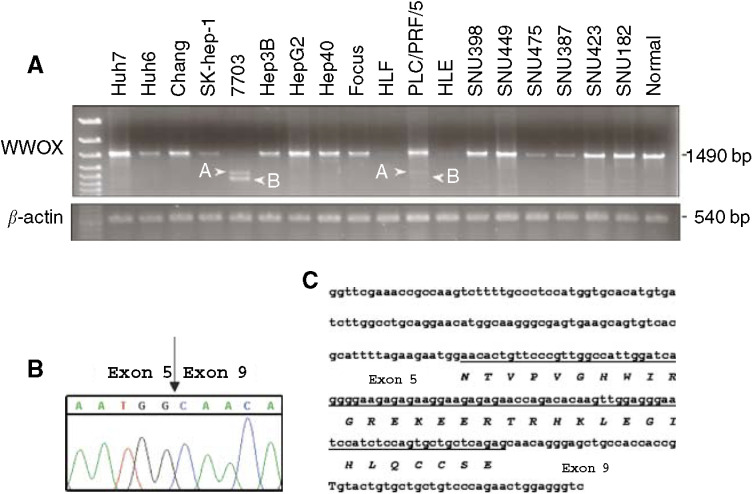
RT–PCR analysis of full-length open reading frame of the WWOX cDNA and detection of aberrant transcripts in HCC cell lines. (**A**) PCR amplification of HCC cell line cDNAs. The 1490-bp product representing the complete open reading frame of the wild-type WWOX transcript was seen in 15 HCC cell lines, whereas only the aberrant transcripts A and B were seen in the 7703 cell line. The PLC/PRF/5 cell line showed the two smaller amplification products in addition to normal PCR product (1490 bp). The amplification of *β*-actin was shown as a positive control for cDNA synthesis. (**B**) Sequence analysis of the two aberrant WWOX transcripts in the 7703 and PLC/PRF/5 cell lines. Fragment A lacked exons 6–8 and contained an intronic DNA insertion of 96 bp. In fragment B, exons 6–8 were deleted. Arrow indicates abnormal fusion between exon 5 and exon 9. (**C**) DNA sequence of the newly identified WWOX splice variant. The partial sequence of WWOX exons 5 and 9 is shown and the 96-bp insertion is underlined. The predicted 32 amino-acid sequence of the novel WWOX variant is given, showing the in-frame fusion with the sequence encoded by the insertion (underlined).

The expression of WWOX mRNA in HCC cell lines was examined by Northern blotting. The 2.2-kb WWOX mRNA transcript was detected in normal human liver, but a decreased level or absence of WWOX expression was observed in 11 out of 18 HCC cell lines. In SK-Hep-1, 7703, HLF and HLE cells, WWOX mRNA was undetectable and in Huh6, Chang, Hep3B, Focus, SNU423 SNU475, and SNU387 cells, WWOX was significantly underexpressed. Only one cell line, HepG2, showed an elevated level of the WWOX transcript (Figure 2

). The cell lines were also examined by RT–PCR, and the expected product of 1490-bp, spanning exons 1–9 of the WWOX gene, was observed in 15 cell lines and in normal liver tissue. In 7703 cells, two shorter transcripts were detected and sequencing revealed that the shortest PCR product was the WWOX Δ6–8 isoform (Bednarek *et al*, 2001; Paige *et al*, 2001; Driouch *et al*, 2002) lacking exons 6–8 (Figure 3A and B

). The larger PCR product lacked exons 6–8 but also contained a 96-bp sequence inserted between exons 5 and 9, identifying a new WWOX transcript variant (Figure 3C). The insert sequence is encoded by nts. 5281331–5281425 (NCBI MapViewer build 34 version 2) of chromosome 16 located within WWOX intron 8. There are no translational termination codons in the inserted sequence, and since the WWOX translational reading frame is maintained, the transcript can potentially encode an approximately 30 kDa protein. The PLC/PRF/5 cell line also showed smaller amplification products in addition to the normal 1490-bp PCR product. Sequence analysis of the shorter bands revealed that they displayed the same alterations, deletion of exons 6–8 with 96-bp insertion and deletion of exons 6–8 alone, as in the 7703 cell line (not shown).

### Expression of WWOX protein

**Figure 4:**
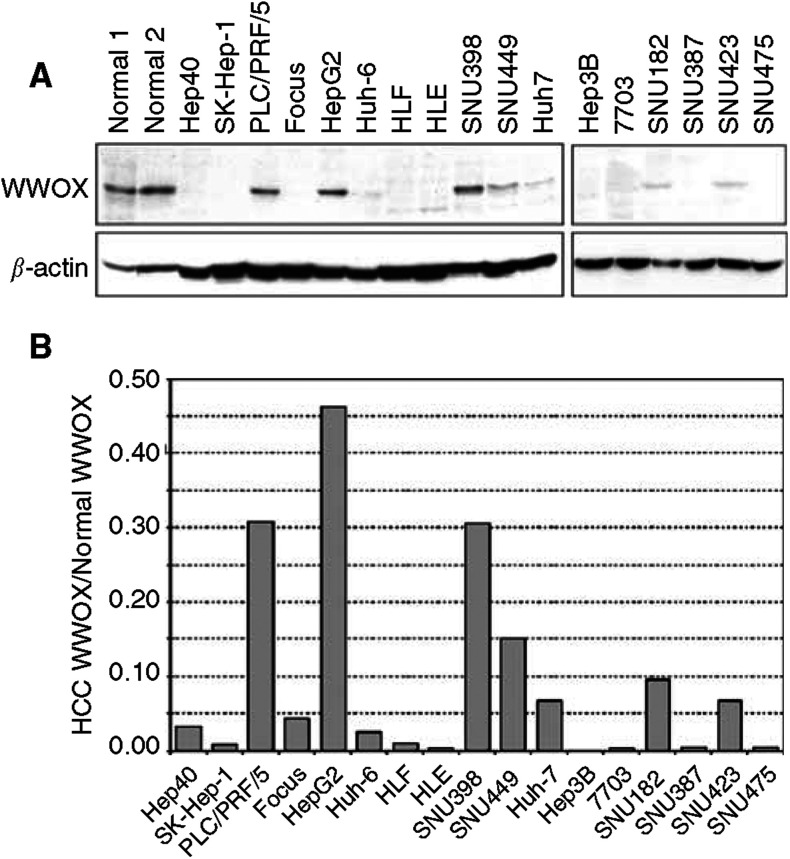
Western blot analysis of WWOX protein in HCCs. (**A**) WWOX polyclonal antibody was used to detect WWOX protein in total protein extracts. Two normal liver samples (normal 1 and normal 2) were used to determine WWOX levels in nonmalignant tissue. Protein loading was normalised by Western analysis of actin levels using a mouse antiactin antibody. (**B**) Densitometric measurements from Western blots were used to calculate WWOX protein levels in HCC cell lines relative to WWOX protein levels in normal liver (WWOX HCC/WWOX normal).

To determine whether WWOX protein expression was altered in HCC, we performed Western blot analysis of lysates from the HCC cell lines and from normal liver tissue. We detected WWOX using affinity purified polyclonal antibody raised against WWOX amino-acid residues 12–94, containing both of the WW domains. This antibody has been used to detect WWOX protein expression in several breast cancer cell lines, demonstrating that altered WWOX gene expression correlated with altered WWOX protein levels (Ludes-Meyers *et al*, 2003). Each normal liver sample had an easily detectable 46 kDa protein band corresponding to WWOX (Figure 4A

). Western analysis revealed that WWOX protein levels in HCCs varied dramatically compared to normal liver (Figure 4A) and, with the exception of Hep40 and Hep3B, WWOX mRNA levels correlated with WWOX protein levels. All of the HCCs had >50% reduction of WWOX levels compared to normal liver when normalised to actin (Figure 4B). The majority of cell lines, 72% (13 out of 18), had WWOX expression levels lower than 90% that of normal liver.

**Figure 5:**
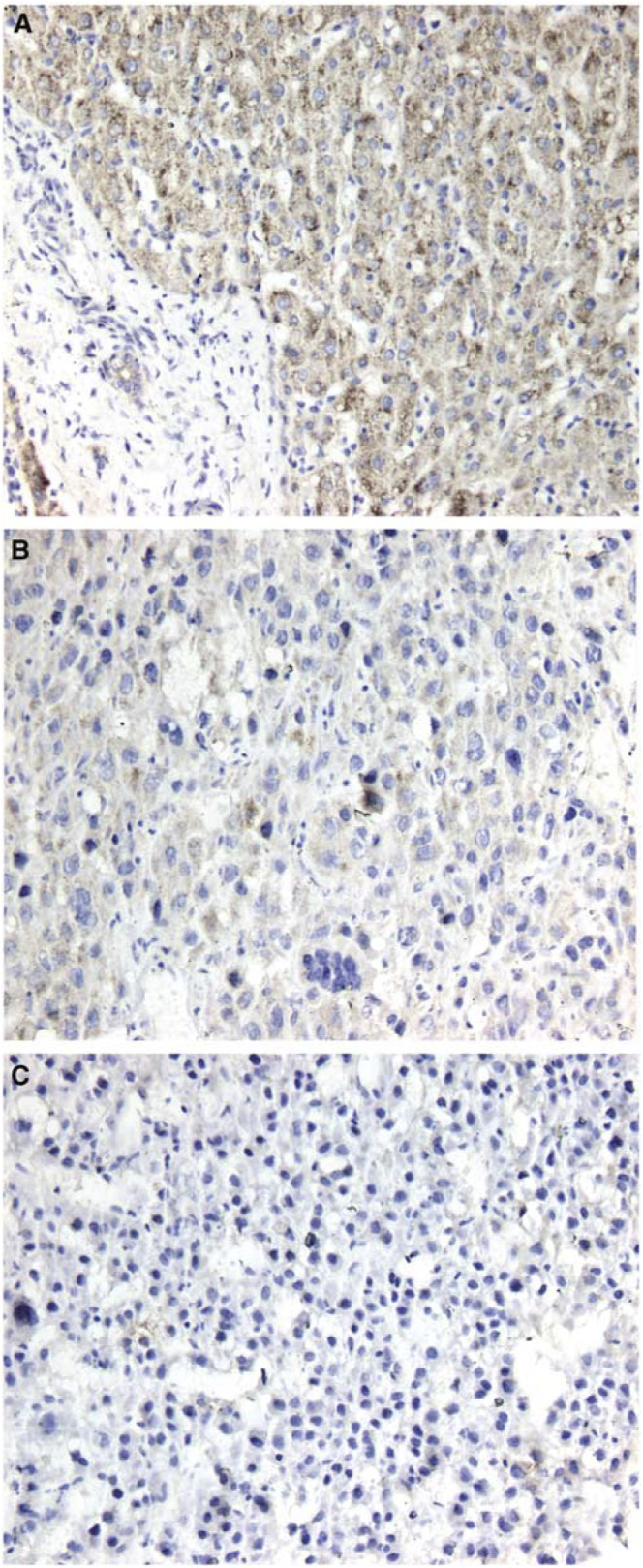
Immunohistochemical detection of WWOX protein in normal liver and HCC. Representative immunostaining results of WWOX protein expression. (**A**) Normal liver tissue sample. Note the strong WWOX cytoplasmic staining of hepatocytes contrasting with the negative staining of the stromal components (lower left corner). (**B**) HCC tissue sample demonstrating very weak WWOX staining. (**C**) HCC sample negative for WWOX staining.

WWOX expression in normal liver tissue was also confirmed by immunohistochemistry. Hepatocytes from five normal liver displayed a strong cytoplasmic staining for WWOX protein as shown in Figure 5A

. Of five primary HCC cases analysed by immunohistochemistry, two of the HCC samples stained very weakly or moderately for WWOX while one sample was negative for WWOX staining as shown in Figure 5B and C, respectively.

### WWOX gene sequence variants

To determine the incidence of somatic mutations of WWOX in HCC, PCR-SSCP analysis of all exons was performed in the 18 HCC cell lines. Direct sequencing of the bands showing mobility shifts revealed that they were due to four previously described nucleotide alterations in the WWOX coding region. The first variation involved a G to A transition at nucleotide 547 that results in an aspartic acid to asparagine substitution at amino acid 183 (GAC to AAC) in 7703 and Focus cell lines. A G to A transition at nucleotide 535, which has been detected previously as frequent polymorphism, was found in amino acid 179 (GCA to ACA) in HepG2 and Hep40, resulting in an alanine to threonine variation. The third variation was detected in SNU387, a C to G transversion at nucleotide 754 that replaced proline with alanine in amino acid 252 (CCT to GCT). The fourth variation was observed in PLC/PRF/5 at nucleotide 941 and consisted of a G to A transition that caused a change from arginine to histidine at amino acid 314 (CGC to CAC). None of these changes affect critical residues of the WW domains or the SDR domain making it unlikely that they alter WWOX protein function and very likely they represent common polymorphisms rather than somatic mutations.

### Concurrent WWOX and FHIT aberrant expression in HCC

In some of the HCC lines examined here, downregulation and lack of expression of the FHIT tumour suppressor gene spanning the common FS FRA3B were previously observed (Yuan *et al*, 2000). Interestingly, three of the HCC cell lines that did not express WWOX (SK-Hep-1, 7703, and HLF) also lacked expression of FHIT (Yuan *et al*, 2000) (Table 1

**Table 1:** Expression of WWOX and FHIT gene in 10 HCC cell lines

**Cell line**	**WWOX**	**FHIT**
PLC/PRF/5	+	+
Focus	+	−
HepG2	+	+
Hep3B	+	−
Hep40	+	+
HLE	−	+
HLF	−	−
7703	−	−
SK-Hep-1	−	−
Huh7	+	−

).

## DISCUSSION

In this study we have demonstrated that alteration of WWOX gene expression is common in cell lines derived from human HCC, suggesting that WWOX might be involved in hepatocarcinogenesis. The interest in the WWOX gene is due to its chromosomal location and potential tumour suppressor activity (Ludes-Meyers *et al*, 2003). The WWOX gene encompasses FRA16D, the second most actively expressed FS in humans and a region prone to breakage with an increased risk for carcinogen-induced damage (Popescu, 2003; Ludes-Meyers *et al*, 2003). Susceptibility of FRA16D to carcinogen-induced damage may explain the high frequency of WWOX gene alterations in HCC, a cancer closely associated with exposure to chemical carcinogens and infection with oncogenic viruses (Thorgeirsson and Grisham, 2002).

The data presented here provide evidence for downregulation or absence of WWOX mRNA expression in 61% of the HCC cell lines and a low or undetectable level of WWOX protein in 75% of them. One exception was the HepG2 cell line, expressing the highest level of WWOX and exhibiting an increased DNA copy number on the long arm of chromosome 16 (Figure 1).

In general, our results are in contrast to those of Watanabe *et al* (2003), who concluded that WWOX was expressed in 47 out of 48 cancer cell lines analysed, including the HCC cell lines HLE and HLF, which clearly do not express WWOX RNA and protein by our analysis. Furthermore, in agreement with our results and in contrast to those of Watanabe *et al* (2003), and supporting a putative tumour suppressor function for WWOX, loss of expression at RNA and protein level has been clearly demonstrated in breast, ovarian carcinoma cell lines and in various haematologic malignancies by several research groups (Bednarek *et al*, 2000; Paige *et al*, 2001; Ludes-Meyers *et al*, 2003; Ishii *et al*, 2003).

Downregulation or absence of WWOX mRNA expression in various types of cancer can be attributed to mutation, homozygous deletion, chromosomal translocations within or near the WWOX locus, hypermethylation of the promoter region or alterations in transcriptional control (Paige *et al*, 2001; Ludes-Meyers *et al*, 2003; Ishii *et al*, 2003). Hemizygous loss could also be of relevance since it could lead to haploinsufficiency, and this abnormality by itself could be of relevance in cancer development. Most of the HCC cell lines carry unbalanced translocations of chromosome 16, with some breakpoints in the vicinity of the WWOX locus, as shown by G-banding and chromosome painting and ongoing spectral karyotyping (Keck *et al*, 1999). Homozygous deletions of WWOX were not detected by Southern blot hybridisation and nearly all the cell lines examined have at least one intact chromosome 16q (not shown). An internal deletion in 7703 cells may be responsible for the shorter transcripts, but their origin in PLC/PRF/5 cells is not known. Although the under-representation of the 16q23 region detected by CGH suggests allelic loss of WWOX, such an alteration would not be sufficient to explain the low levels of WWOX gene expression (Driouch *et al*, 2002). Mutation and deletion in the coding region of WWOX are rather infrequent in various types of cancer including HCC (Yakicier *et al*, 2001). As transcriptional silencing or downregulation of WWOX was found to be associated with aberrant DNA methylation and histone acetylation in two leukemia cell lines (Ishii *et al*, 2003), we examined several HCC cell lines expressing a very low level of WWOX mRNA after combined treatment with 5-aza-2′-deoxycytidine, inhibitor of DNA methyltransferase and trichostatin A, a histone deacetylase inhibitor. Consistent with previous results in breast tumour cell lines, the combined treatment did not increase the level of WWOX mRNA expression (not shown), suggesting that hypermethylation of the WWOX gene promoter might not be common in HCC (Bednarek *et al*, 2001). The low levels of WWOX protein in the majority of the cell lines could also be due to post-transcriptional regulation. The Western blot results were further supported by preliminary immunostaining observations. All normal liver tissue samples stained strongly for WWOX protein while some HCC samples displayed weak or undetectable WWOX immunostaining (Figure 5). Further investigations should be directed toward a better understanding of the transcriptional and translational control of WWOX gene expression.

An important observation is the association of WWOX and FHIT alterations in a number of HCC cell lines. A similar association of WWOX and FHIT alterations has been reported in haematopoietic tumours (Ishii *et al*, 2003). Also, the expression analysis of 45 common apc-induced FSs in 20 karyotypically normal individuals showed that only FRA3B and FRA16D were expressed in all individuals (Denison *et al*, 2003). It has been known for some time that FSs replicate late during the cell cycle, but only more recently the critical role of replication in the expression of FSs has been demonstrated (Laird *et al*, 1987). The replication pattern of FRA3B and FRA16D sequences strongly suggested that late replication or delayed replication progression is a determinant factor in FSs expression (Le Beau *et al*, 1998; Palakodeti *et al*, 2004). A model has been proposed in which spontaneous breaks in ATR-deficient cells represent single-stranded lesions of unreplicated DNA sequences in G_2_ resulting from stalled forks that escape ATR-controlled G_2_/M checkpoint (Casper *et al*, 2002; Arlt *et al*, 2003). ATR as well as BRCA1 are key checkpoint genes implicated in genome stability (Casper *et al*, 2002; Turner *et al*, 2002). This raises the possibility that breast tumours derived from patients with BRCA1 mutation might have a higher rate of WWOX alterations. As common FSs are susceptible to and preferentially targeted by the same carcinogenic agents, it is conceivable that breakage at WWOX/FRA16D and FHIT/FRA3B loci can be inflicted concordantly. Current results with HCC strongly support the notion that alterations of genes spanning FSs are the consequence of carcinogen exposure (Croce *et al*, 1999; Kuroki *et al*, 2002). Alterations of the FHIT gene occur early in tumour development, particularly in cancers related to environmental carcinogens, raising the possibility that carcinogen-induced alterations of WWOX gene are also an early event in the process of hepatocarcinogenesis (Yendamuri *et al*, 2003). WWOX alterations are common in squamous cell carcinoma of the lung and oesophageal cancer, both of which are related to tobacco smoke and alcohol consumption (Kuroki *et al*, 2002; Yendamuri *et al*, 2003). Homozygous deletions at FRA16D have been identified in HCC derived from patients exposed to aflatoxin B1 (Yakicier *et al*, 2001). Interestingly, the 7703 HCC cell line that lacks WWOX protein expression and expresses only aberrant WWOX transcripts was established from a patient in Qidong, China, where viral infection and dietary aflatoxin exposure of the local population are major cooperating risk factors in the development of HCC (Ming *et al*, 2002). HBV infection may not be a contributing factor in loss of WWOX expression, as five of six HBV-negative HCC cell lines expressed a low level of WWOX mRNA and protein. Recently, a functional interaction between p73 and WWOX protein resulting in redistribution of nuclear p73 to the cytoplasm and the suppression of p73 transcriptional activity was demonstrated (Aqeilan *et al*, 2004). Understanding the interations and pathways involved in the tumour suppressor function of WWOX may provide new targets for therapy of various cancers including HCC.
